# Improving Timely Access to Diagnostic and Treatment Services for Lung Cancer Patients in KwaZulu-Natal, South Africa: Priority-Setting through Nominal Group Techniques

**DOI:** 10.3390/ijerph19041918

**Published:** 2022-02-09

**Authors:** Buhle Lubuzo, Khumbulani W. Hlongwana, Themba G. Ginindza

**Affiliations:** Discipline of Public Health Medicine, School of Nursing and Public Health, University of KwaZulu-Natal, Durban 4001, South Africa; hlongwanak@ukzn.ac.za (K.W.H.); ginindza@ukzn.ac.za (T.G.G.)

**Keywords:** lung cancer, South Africa, cancer care, barriers, consensus methods

## Abstract

Background: Lung cancer is the most common cancer worldwide, and it disproportionately affects low-income countries (LICs), where over 58% of cases occur. It is an important public health concern, given its poor healthcare outcomes, yet it is under-researched compared to other cancers. Lung cancer is also very difficult for primary care physicians to diagnose. In many settings, health researchers and clinicians’ resort to engaging in collaborative efforts to determine the best way to implement evidence into routine clinical practice. Methods: This was a grounded theory study comprising seven experts providing oncological services. A Nominal Group Technique (NGT) was used to articulate ideas, identify key problems and reach consensus on the order of priorities for the identified problems. Results: The study findings revealed that access to healthcare facilities providing oncology services and diagnosis was the major barrier to lung cancer care. This was further exacerbated by the manner in which health systems are configured in South Africa. The priorities for the health providers were focused on the lack of specialized resources, whereby referral of patients suspected to have lung cancer was delayed and compounded by the limited availability of treatment. Conclusion: The inadequacy of supportive systems for access to healthcare services negates the government efforts to curb the rising lung cancer-related fatalities in South Africa.

## 1. Introduction

Cancer is currently responsible for more than 7 million deaths per year worldwide [1]. If the current trend is not averted, over 20 million new cancer cases across the globe are projected for 2025, compared to about 14.1 million and 17.5 million new cases in 2012 and 2015, respectively [2,3,4,5]. Of these cases, lung cancer remains the leading cause of cancer-related deaths globally [6]. In both high-income countries (HICs) and low- and middle-income countries (LMICs), lung cancer accounted for 1.6 million cancer-related deaths annually (approximately 20% of total cancer deaths), with an estimated 1.8 million new annual cases worldwide [3]. Evidence suggests that, by 2030, an estimated 85% increase will be observed in sub-Saharan Africa (SSA) [7].

Lung cancer is the leading cause of years of life lost and it is associated with the highest economic burden relative to other tumor types [8]. Research remains central to implementing evidence-based interventions and improving health outcomes in lung cancer. While lung cancer is the most common cancer worldwide, it disproportionately affects low-income countries (LICs), where over 58% of cases occur [9]. Africa’s reported low incidence rate of lung cancers (7.7 per 100,000 in men and 2.6 per 100,000 in women, respectively) is attributable to the critical lack of accurate data, reflecting enormous underestimations of the true lung cancer burden in the continent [10]. Accordingly, it is essential to know the magnitude of lung cancer and its implications in different regions in Africa through establishing functional cancer registries.

The burden of lung cancer is growing in South Africa (SA) and imposes strain on health infrastructure and the allocation of already limited resources [11]. The growing burden of lung cancer in the country is attributable to several reasons, including the absence of a cost-effective screening tool, diagnosis at advanced stages (approximately 80% of cancer patients are diagnosed at stages 3 and 4) and socio-economic inequalities in health care access [12,13,14,15,16,17,18,19,20,21,22,23,24,25,26,27,28,29,30,31,32,33,34,35,36,37,38,39,40,41]. Unless the situation is substantially changed through various interventions, including health education and behavior change programs, the health systems will soon barely be able to cope with the cancer burden [42,43]. There is increasing recognition that change should start at the health systems level, such as policy implementation, financing, educational reform, and strengthening of leadership, management, and governance [44].

**Figure 1:**
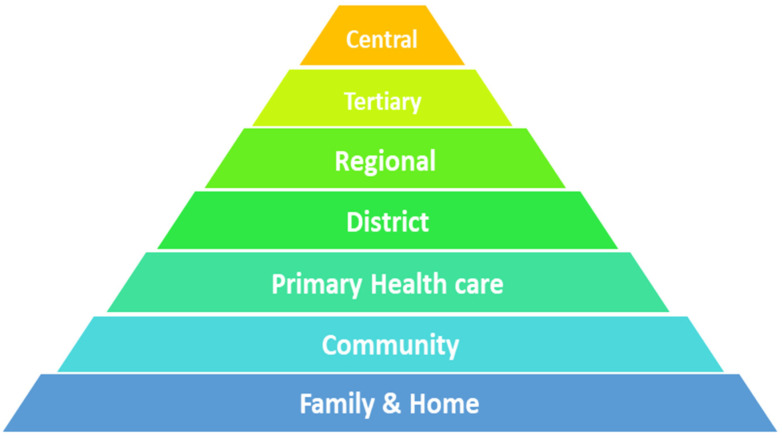
Organizational and Referral hierarchy of oncology and general health services in KZN Source: Kutzin & Barnum [45].

The health service structure in SA, and in KwaZulu-Natal (KZN) province in particular, follows a pyramidal approach (Figure 1) with the first formal referral expected to begin at the primary level, progressing to the secondary level, tertiary level, quaternary levels and medical training institutions [45]. The healthcare provided at primary healthcare clinics is of low quality, owing to limitations pertaining to infrastructure and expertise. This, in turn, prolongs the time to receiving specialized patient care available at tertiary and quaternary facilities [19,21,23,24,29,30,31,34,36,40,41]. These bottlenecks often result in deaths before patients’ access to tertiary hospitals, where advanced diagnostic procedures and treatments are administered [45,46]. Barnum and Kutzin’s illustration indicates the hierarchy of access to health care [45]. The bottom part of the pyramid refers to home-based care. Individuals take care of their own health assisted by and through the support of community caregivers for preventative and promotive health and for disease screening and monitoring [20]. From this point, if the health condition warrants, the patient is referred to a primary health care facility and referred stepwise to other levels of care. In theory, this referral pathway ensures that patients receive care appropriate to their needs in the most cost-effective way. However, the downside to it may be the delay in getting cancer patients diagnosed and treated in a timely manner [45,46]. Whereas stage at diagnosis is a strong predictor of survival, delaying the referral pathway could increase the risk of stage progression and poorer clinical outcomes [12,15,16,19,21,22,23,26,29,30,34,35,41,47].

The illustration indicates the hierarchy of access to health care services. Access relates to the opportunity to obtain and appropriately use quality health services. It is concerned with the “degree of fit” or compatibility between the health system on the one hand and individuals who need to use these services on the other hand. The availability dimension of access deals with whether the appropriate health services are available in the right place and at the right time to meet the needs of the population.

Identification of the current barriers to and priorities for effective lung cancer care coordination is needed for healthcare improvement purposes, and studies investigating the stakeholders’ perspectives, including health professionals, involved in the delivery of cancer healthcare services are rare. Lung cancer is under researched compared with other cancers [48] and is one of the most difficult cancers for primary care physicians to diagnose [49,50]. Setting priorities in health services delivery and research is of great relevance to ensuring the optimal use of scarce resources. Therefore, the aim of this study was to investigate health providers’ perspectives on barriers and priorities to lung cancer patient access, diagnosis, referral and treatment in three public health facilities providing oncological services in KZN, SA.

## 2. Materials and Methods

### 2.1. Study Area

The study area was Durban (DBN) and Pietermaritzburg (PMB), located in the eThekwini and uMgungundlovu District Municipalities, respectively, and these are the two most populous districts in KZN Province. KZN is the second most populous province in SA, with a total population of 11.4 million people (19.7% of SA total population) [23]. In 2014, Gauteng and KZN, the first and second most populous provinces in SA, respectively, accounted for the highest number of cancer deaths, with lung cancer having the highest rates in Gauteng and second highest in KZN, respectively [23].

### 2.2. Study Setting

This study was conducted among the healthcare professionals from the three health facilities providing oncology services in KZN. These facilities are Greys Hospital located in PMB, and Addington Hospital and Inkosi Albert Luthuli Central Hospital (IALCH) located in DBN. DBN and PMB are the biggest cities in KZN [51]. The health facilities were chosen on the basis of their being the only public hospitals offering oncology services in the province.

### 2.3. Study Design

**Figure 2:**
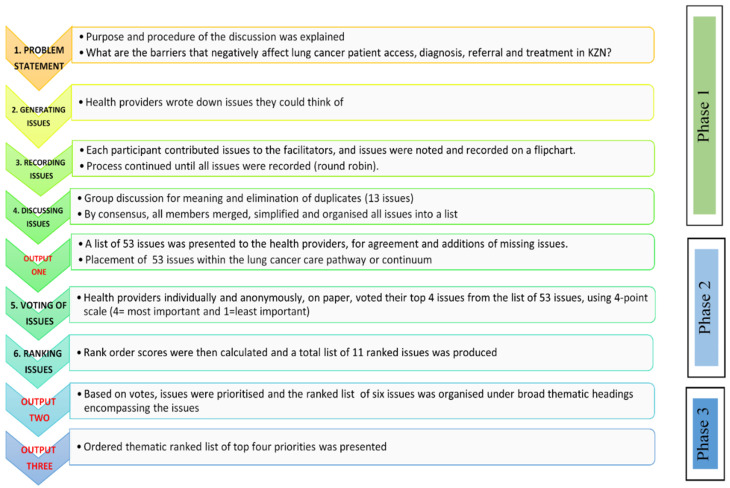
Nominal Group Process Flow followed in this study. Source: Developed from this study.

This study used the Grounded theory (GT) design, which is widely used in health and social sciences to generate theoretical accounts of social phenomena; this approach is appropriate when research aims to explain a process where the concerns of those involved are central to its understanding and cannot be predetermined. A Nominal group technique (NGT) was used to identify and prioritize the key issues from the health providers’ perspectives in KZN. The interpretive approach followed in NGT’s interactive discussions with stakeholders to generate priorities [52,53,54,55] produced both quantitative and qualitative data. NGT has demonstrated validity, and it emphasizes considering all participants’ views equally and enables consensus on highly complex issues [52,53]. The study followed all six stages of NGT implementation [52,53] (Figure 2), covering three outputs, namely: a list of 53 issues placed within the lung cancer care pathway or continuum; listing and voting for the final judgments on issues; and an ordered thematic ranked list of the top four priorities was presented.

This article stems from a study that broadly investigated healthcare providers’ perspectives on the barriers to providing lung cancer patients with quality care in KZN. Eighteen healthcare professionals were interviewed in order to explore these barriers. A further subset of seven healthcare professionals were invited for NGT discussions based on the richness of information shared during the in-depth interviews (IDIs). NGT discussions built on the barriers identified during the IDIs and these barriers were further subjected to a priority-setting exercise. The results from IDIs showed that inadequate knowledge and expertise of patients and practitioners in public primary healthcare clinics were the major sources of the diagnosis-related barriers, suggesting inadequate training of healthcare professionals at lower-level facilities. Healthcare professionals participating in IDIs contended that people with lung cancer typically fail to act quickly enough because they ascribe the symptoms to less serious causes or simply lack sufficient knowledge to identify lung cancer symptoms. This article focuses on the results of the NGT discussion after the ranking exercise of the barriers to lung cancer care.

### 2.4. Study Population and Sampling Strategy

The targeted panel of experts providing oncological services, referred to as healthcare providers, was recruited on the basis of current knowledge and perceptions on the research subject. The study participants considered to be information-rich were purposively selected, and this was guided by their specialties, experiences [56] and their inputs during IDIs. Seven participants were considered adequate to achieve the objectives of the study and this is supported by the literature [57]. Seven of the eight identified potential respondents accepted the invitation and participated in the NGT discussion and this group size is supported by the literature [54]. All participants signed informed consent forms prior to their participation.

### 2.5. Data Collection and Analysis

The structured group discussion for NGT started off with a researcher’s presentation of the preliminary results from the individual IDIs, given that almost all of them had participated in the IDI component of the study. Subsequently, an adapted NGT stepwise process flow was followed (Figure 2). A number of issues were raised during the presentation and these issues were deferred to the NGT process. NGT started with the presentation of the following problem statement:

*“Cancer cases, especially lung cancer, are often detected late, which in turn affects patient survivorship. In most instances, and as evidenced by the preliminary findings from the in-depth interviews the Principal Investigator presented, the late detection and survivorship challenges can be attributed to barriers related to the pathways of cancer care.*

*Kindly list all the barriers that you can think of, which negatively affect lung cancer patient access, diagnosis, referral and treatment in KwaZulu-Natal”.*

Phase 1: Following from the above problem statement, group members were asked to silently brainstorm and come up with all possible issues or barriers to lung cancer care and to make a note of each on a separate note page after they had been provided with the necessary stationery. All issues were put in a box and each individual randomly took five issues, which they read aloud for the facilitator to record on a flipchart. Each flipchart sheet was taped on the wall for all to read and see. As a group, we discussed, questioned and clarified the issues. We also individually evaluated the shared ideas.

Phase 2: The participants anonymously voted for the top four key issues. Based on votes, issues were prioritized, and the ranked list was organized under broad thematic headings encompassing the issues. Individuals voted privately to prioritize issues, using moderator-created criteria. The criteria were described by the facilitator, and the participants had an opportunity to seek any clarification before privately rating each issue. Participants were then asked to rate each issue across the criteria listed above without sharing these with others. This was considered an important step as this private rating of items allowed participants to make their own considered judgements prior to reaching group consensus.

Phase 3: Lastly, we shared votes and a report was prepared showing the ideas receiving the most points. Briefly, we then allowed time for informal individual views on solutions they raised with regard to the four key issues or barriers to lung cancer patient access, referral, diagnosis, treatment and care. The ranked list and notes from the NGT discussions were collated electronically using a Word document while consulting facilitator session notes and recording to explain aspects that needed further clarification. Thereafter, we calculated the ratings and recorded the cumulative rating for each idea.

The diagram presents a summary of the NGT process, which includes a number of steps that we adapted while retaining the goal of the NGT method. NGT implementation involves six stages. It began by allowing members of the group to individually brainstorm, which is a fact-finding and idea generation stage. The second stage is more structured with group members coming together to exchange and/or interact regarding the ideas each member generated, and the final stage involves listing and voting for the final judgments of ideas. Ideas are then ranked in the order of priorities (Table 1).

### 2.6. Ethical Consideration

The study obtained the ethics approval and gatekeeper permission from the University’s Ethics Committee (Ref: BE332/18) and KwaZulu-Natal Provincial Department of Health (HRKM Ref: 007/18 and NHRD Ref: KZ_201801_031). All three participating health facilities supported the study. Selected healthcare providers voluntarily signed informed consent forms prior to participating in the study. Confidentiality was maintained through anonymizing their contributions in all written materials.

## 3. Results

The NGT panelists comprised three oncologists, a radiotherapist, pulmonologist, social worker and a member of a cancer hospice non-governmental organization. Five of the seven participants had participated in our IDI data collection stage.

### 3.1. Phase One

**Figure 3:**
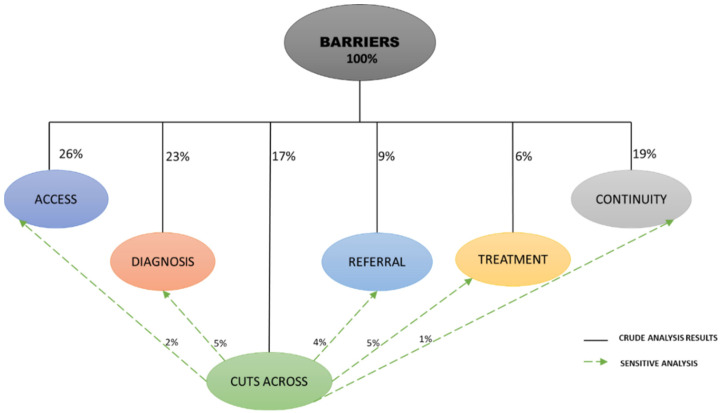
Mapping the proportion of barriers within lung cancer care continuum (Phase 1).

**Figure 4:**
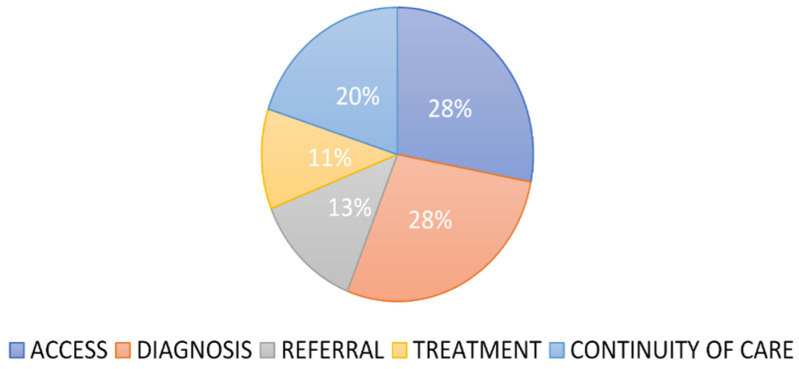
Distribution of the barriers within lung cancer care continuum.

Stages one to three comprised the brainstorming and recording of key issues, which generated a total of 53 barriers for prioritization. These were further placed within the care continuum (Figure 3). Of the 53 barriers, 26% (*n* = 14) were placed under the access point of the lung cancer care continuum, followed by diagnosis point with 23% (*n* = 12), while treatment had only 6%. Sensitive analysis was further carried out to assess the proportion of the ‘cutting across’ barriers, constituting 17% (*n* = 9) of the total barriers. Using the sensitive analysis, we then distributed the issues within the identified points of the lung cancer care continuum (Figure 3). After adding the sensitive analysis results to crude analysis, both access and diagnosis became the top barriers to lung cancer care continuum with 28%, followed by continuity and supportive care with 20% (Figure 4).

Figure 3 shows the sensitive analysis of the barriers which were cutting across the points of care within the continuum.

Figure 4 shows the distribution of the barriers within the lung cancer care continuum points after the calculation of sensitive analysis shown in Figure 3.

### 3.2. Phase Two

After stage four, where duplicates were eliminated and issues were clarified, a final list of 40 barriers were identified and recorded on a flipchart. Some of the key issues shared by the health providers were the ‘lack of screening services’, ‘ill-informed practitioners at first consultation or investigation point’, ‘rushed healthcare workers’ and ‘poor documentation by the multidisciplinary team’. Other issues highly rated by the participants were ‘lack of coordinated care throughout the lung cancer care continuum by the multidisciplinary team’, ‘limited access to trained personnel’, ‘delays at first consultation stage’ and ‘direct and/or indirect costs related to care’.

### 3.3. Phase Three

From the list of barriers, the healthcare providers ranked the four responses or issues most important to them. The most serious issue was allocated 4 votes, the second and third most important allocated 3 and 2, respectively, with the least important being allocated 1 vote. Table 1 presents the 6 issues that were rated the highest by the healthcare providers after being grouped into thematic areas and shared with the participants. The ranking exercise identified ‘lack of specialized resources’ as the leading issue with 26 points, followed by ‘limited community lung cancer awareness’ with 14 points, whereas ‘absence of referral guidelines or policy’ and ‘education and training on lung cancer’ were 3rd and 4th with 12 and 7 points, respectively (Table 1).

In relation to specialized resources, there has also been little progress on changing the way in which financial resources are allocated to promote distribution in line with relative need for health services. The distance people need to travel to a health facility is a key element of the availability dimension of access. The likelihood of using a health service is far lower for those living furthest from health facilities. The promotion of equitable access to quality health care was seen as a priority by the panelists.

The emphasis placed on education and training far exceeded issues such as waiting time and whether care was provided by a nurse or a doctor (i.e., skills mix amongst professionals). Professionals at these lower-level facilities are not sufficiently equipped and trained, resulting in substandard performance in early diagnosis and referral. At the symptom onset point, misclassification of lung cancer as tuberculosis (TB) affects patient access to lung cancer care. Limited lung cancer knowledge appeared to affect patient access both at the health provider and patient levels, resulting in the need for lung cancer awareness. The inadequacy of systems that support access to healthcare services facilitates the rising numbers of lung cancer fatalities in SA. Delays in both presentation and in diagnostic workup negatively impact the effectiveness of the pathways.

## 4. Discussion

Geographic access is an important part of accessing health care in LMICs. An inverse relationship between distance or travel time to health facilities and use of health services has been demonstrated as an important barrier to access. Lack of adequate communication services also limits access to health care. In support, the NGT discussions identified practitioner knowledge and expertise at the lower level of care as one of the top four priorities for improving lung cancer care. All of the above evidence relates to the public health sector in SA. The healthcare providers at the lower levels of care, who are the first access point to patients, are not well equipped and trained, thereby compromising their ability to perform early diagnosis and referral. Following the application of the NGT approach, consensus was achieved within the anonymous rankings, and this enhanced the credibility of the conclusions drawn from the data. Despite the fact that healthcare workers were from different healthcare facilities and professional backgrounds, their rankings of the barriers to lung cancer care were consistent and may be reflective of the overall issues affecting the provision of lung cancer care in KZN.

The burden of cancer in African countries continues to rise and survival rates are still affected by late detection, delay in seeking diagnosis, unavailability of technical support for diagnosis, lack of resources for treatment and the availability of palliative care. Consistent with the manner in which the South African health system is configured (Figure 1) [45], the study findings demonstrated that access and diagnosis points to lung cancer care remain the major barriers, with the cancer diagnostic services only offered at high levels of care [20,58]. Patients are expected to initiate their health-seeking from the primary healthcare clinics before progressing to subsequent levels, a phenomenon that is time consuming and not unique to SA [9,30,46,59,60,61,62]. This phenomenon culminates in patients being diagnosed with cancer at advanced stages [19,21,23,24,29,30,31,34,36,40,41].

Proper diagnosis and access to cancer specialists (health professionals and facilities) is also critical for the management of lung cancer. Based on our findings, access to diagnostic services of lung cancer remains amongst the key public health challenges requiring targeted interventions, including the strengthening/expanding of existing cancer screening in the country with emphasis on the populations at risk (smoker, miners and Tuberculosis (TB) patients). Lobbying and advocating for the development of a lung cancer specific screening policy, as well as the introduction of ongoing lung cancer awareness at the national level, are needed to implement a comprehensive lung cancer prevention program. Success depends in part on gaining a local understanding of the dimensions and determinants of access to health services, along with determined attempts to improve those services.

It is true that most of the limitations found in using qualitative research techniques also reflect their inherent strengths. For example, small sample sizes like the NGT method help the researcher to investigate research problems in a comprehensive and in-depth manner, but the same small sample size also limits the generalizations of findings. Additionally, as the primary instrument of investigation, qualitative researchers are often embedded in the cultures and experiences of others. However, cultural embeddedness increases the opportunity for bias in the manner in which data is gathered, interpreted, and reported. Strengths of this study include representation of a range of views of health professionals with extensive experience in lung cancer patient care across a variety of geographic and health care settings and disciplines in KZN. However, the nature of the sample size for NGT limits, at least in part, the transferability of these perspectives to all health professionals working in lung cancer care, especially since healthcare providers from the private hospitals offering oncology services were not included. The systematic nature of the NGT process, the inclusion of participants from all three health facilities providing oncology services in KZN, and the mix of professionals involved in cancer care were the greatest strengths of this study. However, the non-inclusion of healthcare workers from the lower levels of care remains an important weakness of this study. Since the pathways of care for any health condition begin from the primary healthcare clinics, future studies should consider exploring the perspectives of healthcare workers from the lower levels of care and private health sectors in SA and ensure that such data is maintained on an ongoing basis.

The NGT is a highly adaptable method and can be used in addition to, or to inform other methods. It begins with allowing members of the group to individually brainstorm, the stage that Boddy refers to as fact-finding and idea generation [52,53,54,55]. The second stage is more structured with group members coming together to exchange and/or interact regarding the ideas each member generated, and the final stage involves voting for the final judgments of ideas. Ideas are then ranked in the order of priorities [52,53].

Studies that report on findings using NGT are rare; hence, this article provides an important contribution to the field. The quality and reliability of services provided at lower-level hospitals depend on the efficiency of the functioning of a health care system. If patients have doubt or do not trust the quality of services they receive at the lower levels, they may bypass them and refer themselves to functioning higher levels. This leads to inappropriate utilization and inefficiencies in the health care services.

## 5. Conclusions

The NGT was efficient in raising a large number of issues affecting patient access, diagnosis, referral and treatment of lung cancer in KZN. Although all the issues generated were important to this group of healthcare providers, the top six issues were considered top priority. This is anticipated to assist in developing appropriate intervention strategies as well as policy guidelines and informing policy makers. These health providers are, in one way or another, involved in cancer care and understanding their experiences and priorities would be a key step towards improving care. Addressing these issues could prove particularly useful to improving support for the important work of these specialists who are providing oncology services in KZN. Some of the priorities identified in this study require financial resources, which were also listed as an important issue affecting lung cancer management in KZN. Investing in resources and good patient referral systems could improve timely, coordinated, continual and consistent lung cancer management. As supported by the findings, personnel from the lower levels of care require education, training and equipping to be able to efficiently provide lung cancer screening and diagnosis and treatment. Finally, the results of this study can be used by advocacy groups to make a case for improved lung cancer care in KZN in particular and SA in general. Additionally, the robustness of the NGT makes the results of this study somewhat transferable to settings with comparable characteristics and similar challenges.

## Figures and Tables

**Table 1:** Top Priority Issues as Identified by NGT Session of Healthcare Providers (Phase 2 & 3).

Key Issues	Respondent 1	Respondent 2	Respondent 3	Respondent 4	Respondent 5	Respondent 6	Respondent 7	Points	Ranking
**A-Specialized Resources**	4	3	4	4	3	4	4	26	1st
**B-Screening Services**		4		2				6	-
**C-Awareness**	2	1	2		4	2	3	14	2nd
**D-Referral Guidelines**	3		3	3	2	1		12	3rd
**E-Education & Training**	1	2		1		3		7	4th
**F-Co-ordinated Care Plan**			1		1		2	4	-
**G-Financial Constraints**							1	1	-

**A** = Lack of specialized resources| **B** = Limited screening services| **C** = Community lung cancer awareness| **D** = Absence of referral guidelines/protocols| **E** = Education & training (practitioner knowledge and expertise) | **F** = Lack of coordinated care plan by multidisciplinary team| **G** = Financial constraints.

## Data Availability

Data from this study are the property of the KwaZulu-Natal Department of Health and University of KwaZulu-Natal (UKZN) and cannot be made publicly available. All interested readers can access the data set from the UKZN Biomedical Research Ethics Committee (BREC) from the following contacts: The Chairperson Biomedical Research Ethics Administration Research Office, Westville Campus, Govan Mbeki Building University of KwaZulu-Natal P/Bag X54001, Durban, 4000 KwaZulu-Natal, South Africa, Email: BREC@ukzn.ac.za; Tel.: +27-31-260-4769; Fax: +27-31-260-4609.
